# A Collaborative Implementation Strategy to Increase Falls Prevention Training Using the Age-Friendly Health Systems Approach

**DOI:** 10.3390/ijerph19105903

**Published:** 2022-05-12

**Authors:** Jennifer Jurado Severance, Solymar Rivera, Jinmyoung Cho, Jessica Hartos, Amal Khan, Janice Knebl

**Affiliations:** 1 Department of Internal Medicine and Geriatrics, University of North Texas Health Science Center, Fort Worth, TX 76107, USA; amalkhan@my.unthsc.edu (A.K.); janice.knebl@unthsc.edu (J.K.); 2 Department of Rehabilitation and Health Services Research, University of North Texas, Denton, TX 76203, USA; solymarrivera@my.unt.edu; 3 Center for Applied Health Research, Baylor Scott & White Research Institute, Temple, TX 76502, USA; jinmyoung.cho@bswhealth.org; 4 Cizik School of Nursing, The University of Texas Health Science Center at Houston, Houston, TX 77030, USA; jessica.hartos@uth.tmc.edu

**Keywords:** fall prevention, aging, Age-Friendly Health Systems, mobility, falls efficacy

## Abstract

Falls in the home and in community environments are the leading cause of injuries and long-term disabilities for the aging population. The purpose of this study was to examine outcomes of a partnership among an academic institution, government agency, community organizations, and emergency management services to implement a falls prevention training program using an Age-Friendly Health Systems approach. In this prospective study, partners identified gaps in services and targeted and non-targeted delivery areas for implementation of an evidence-based falls prevention intervention addressing the 4Ms of Age-Friendly Health Systems—Mobility, Medications, Mentation, and What Matters. Descriptive statistics were calculated for program implementation and participant demographic variables, and paired *t*-test analysis compared scores for self-assessed general health and falls efficacy prior to and after program participation. Twenty-seven falls prevention classes were implemented, with over half (52%) in targeted areas. A total of 354 adults aged 50 and older participated, with N = 188 participants (53%) completing the program by attending at least five of eight sessions. Of completers, 35% resided in targeted areas. The results showed a statistically significant improvement in falls efficacy by program completers in targeted and non-targeted areas. However, there was no statistically significant difference in self-rated health. Overall, the findings of this study indicate that collaboration to deliver falls prevention training can be effective in reaching at-risk older adults. By mobilizing collaborative partnerships, limited resources can be allocated towards identifying at-risk older adults and improving community-based falls prevention education.

## 1. Introduction

By 2050, the older adult population is projected to double in size to comprise 22% of the global population [1], and unfortunately, most will need to manage chronic conditions, maintain healthy behaviors, and reduce safety risks, such as falls and fall-related injuries, that threaten independence and well-being. According to the World Health Organization (WHO), falls are a major public health concern, with older adults aged 60 and over having the highest risk of falls-related injury and death [2]. The cost of falls increases as a result of injury, functional decline, and disability requiring hospitalization, long-term services, and/or institutionalization. In 2018, three million nonfatal fall-related injuries among older adults aged 65 and older were treated in Emergency Departments (ED) in the United States, and more than 950,000 of these patients were hospitalized [3]. Falls among older adults in the United States cost USD 50 billion for non-fatal fall injuries and USD 754 million for fatal falls each year [4]. The total economic costs of falls will place demands on health systems as the worldwide demographic shift continues.

While the risk of falls and injury increases with age, falls prevention is viable. Falls in older age involve a confluence of modifiable social, behavioral, and physical risk factors, such as decreased strength; impaired vision, balance, and gait; and polypharmacy; as well as hazards of the physical environment, such as poor lighting or slippery floors [5,6]. These and other individual and environmental factors can be mitigated through falls risk assessments and tailored, multifaceted interventions aimed at improving mobility; reducing fears of falling; increasing balance, gait, and strength; eliminating the inappropriate use of high-risk medications; and creating a safe home environment [7,8]. While there is evidence that community-based interventions aimed at multifactorial risks factors can be effective in reducing falls for community-dwelling older adults [9,10], this has not prompted widespread use of community-based interventions to meet the public health need [11,12].

Implementation strategies to increase access to evidence-based interventions for at-risk populations are critical to improve older adult population safety and well-being [13,14,15,16]. With widespread public health challenges related to an aging population, the Age-Friendly framework may be a useful strategy to bring together community partners to help increase the use and improve the implementation of evidence-based interventions in communities [17]. The Age-Friendly Health Systems (AFHS) initiative was created by the Institute for Healthcare Improvement and The John A. Hartford Foundation to improve quality of healthcare for older adults. It provides a guide for developing improvement strategies by focusing on the 4Ms of age-friendly healthcare—Mobility, Mentation, Medication, and What Matters [18]. AFHS provide a systematic strategy for assessing gaps in care for older adults, adopting person-centered processes and evidence-based interventions, aligning care within each of the 4Ms for every older adult patient, and studying performance to improve processes [19]. By applying the AFHS framework to improve mobility care, a health system can consider multilayered aspects of individuals, organizations, and communities to implement evidence-based falls risk interventions and thereby enhance older adults’ ability to continue doing what matters by promoting functional mobility, cognitive health, and medication safety. Researchers have found that integrating the AFHS 4Ms framework has been useful to develop and study quality improvement initiatives within a setting of care, such as in the home, nursing home, hospital, or clinic, to improve patient outcomes in a 4Ms area, such as medication safety or dementia care [20,21,22]. However, this framework should also be used to develop and assess falls prevention strategies at the community-level.

In the United States (U.S.), Texas has the third largest number (over 3 million) of older adults aged 65 and older and the largest proportion (33.6%) of reported falls compared to other states [23,24,25,26]. The risk of falls impacts subpopulations disproportionately, with falls in Texas more likely reported for older adults identifying as Hispanic (34%) or White (34.7%), having an annual income of less than USD 50,000, or having lower educational attainment [26]. Hospitalization-related falls in Texas increase with age and are greater among older adults identifying as female, Hispanic, or White [26]. Texas has one of the highest costs related to falls, with total direct medical costs estimated at over USD 2.4 million in 2014 [23]. With a focus on older adults in Texas, the purpose of this study was to examine outcomes of a partnership among an academic institution, government agency, community non-profit organizations, and emergency management services to implement a falls prevention training program using an AFHS approach to increase falls prevention in North Texas in targeted and non-targeted areas.

## 2. Materials and Methods

### 2.1. Study Design

As part of a U.S. Health Resources and Services Administration’s Geriatric Workforce Enhancement Program (GWEP) grant, the University of North Texas Health Science Center collaborated with a local government agency providing aging services (Area Agency on Aging), community-based organizations delivering evidence-based programs, and an emergency medical service organization for the purpose of expanding access to evidence-based falls prevention training for disadvantaged and high-risk populations in Tarrant County, Texas. Applying an AFHS approach [19], an interdisciplinary team met regularly to develop an implementation plan. The team included clinicians, front-line staff involved with falls prevention training, administrators, and health service researchers from partner organizations. At the beginning of the collaboration, team members shared information to assess gaps in local falls prevention training. Community-based partners provided demographic data to identify low-income underserved older adults in their service population. The emergency medical service organization provided postal code data for falls-related emergency calls for older adults. All organizations provided data about their services and capacity to collaborate in delivering or promoting falls prevention training for older adults. The team used these data to determine service gaps in Tarrant County that predisposed older adults to falls and to identify areas for implementation. The team continued to collaborate monthly to identify challenges and solutions and improve implementation.

Utilizing the growing body of research examining geographical regions such as postal codes zones to identify areas with a high incidence rate of falls among older adults [27,28,29], 13 postal codes with a high number of low-income underserved older adults, a high density of falls-related emergency calls for older adults, and limited prior execution or underutilization of community-based falls prevention interventions were identified as “hot spots” to be considered as “targeted areas” in addition to training that would occur in “non-targeted areas.”

This was a prospective study that followed the recruitment and retention of older adults in the A Matter of Balance (AMOB) training program for falls prevention from July 2019 to June 2020, and assessed within-group changes in health ratings. This study was approved by the IRB of the University of North Texas Health Science Center in Fort Worth (Reference Number: 2018-081).

### 2.2. Intervention

We implemented the A Matter of Balance (AMOB) health promotion program in a twelve-month period through trained volunteer leaders and partnerships with delivery sites (‘implementation sites’) with a goal of determining the enrollment and effectiveness of AMOB classes in targeted and non-targeted areas. AMOB was developed by Boston University’s Roybal Center and is delivered as a weekly or twice weekly class series of eight small group sessions with topics including physical activity, home safety evaluation, identifying and controlling modifiable falls risk factors, and assertiveness [30]. Each session involves group discussion; problem-solving; strength, coordination, and balance exercises; and goal setting [30]. Studies have found that older adults who complete at least five of the eight AMOB sessions report significant improvements in falls efficacy, falls management, and falls control [30,31]. Additionally, AMOB components align with the 4Ms of AFHS as seen in Table 1 [32].

### 2.3. Participants

**Figure 1:**
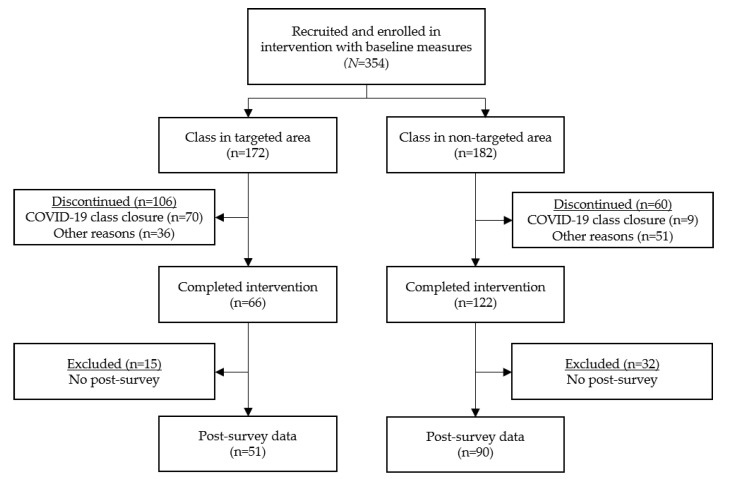
Participant inclusion flow chart.

Participants were recruited through community-based organizations’ websites, flyers, community presentations at retirement communities and senior centers, and word-of-mouth. Participation in the study was voluntary and participants could choose to withdraw from the study at any time. There was no age limit to participate. Twenty-seven AMOB classes were delivered at various implementation sites, including activity centers, independent residential communities, faith organizations, healthcare organizations, and libraries. A total of 354 community-dwelling older adult participants enrolled in classes in targeted and non-targeted areas. Of these participants enrolled, 166 did not complete the program either due to discontinued classes at the onset of the COVID-19 pandemic in March 2020 or due to other reasons. Among those who attended five of eight sessions (n = 188), 141 (75%) completed baseline and post-intervention surveys for inclusion in the study. There were no demographic differences between those who did and did not complete AMOB training. Figure 1 displays a flow chart of the participant inclusion process.

### 2.4. Research Variables

#### 2.4.1. Independent Variables

Program administrative records were used to categorize implementation sites and AMOB classes by targeted versus non-targeted areas (i.e., “hot spot” and “non-hot spot” postal codes), number of participants enrolled, and number attending five of the eight sessions. Participants completed surveys to collect demographic data, including age, sex, race, ethnicity, and primary language.

#### 2.4.2. Dependent Variables

Participants were surveyed for confidence in managing falls using a five-item Falls Efficacy Scale (FES) rated on a 4-point Likert scale at baseline (i.e., at the beginning of the first class) and upon completion of the intervention (i.e., after class 8). The FES was developed by Tennstedt et al. [33] and includes five items: (1) ‘I can find a way to get up if I fall’, (2) ‘I can find a way to reduce falls’, (3) ‘I can protect myself if I fall’, (4) ’I can increase my physical strength’, and (5) ‘I can become more steady on my feet’. Participants rate each item on a scale of 1–4: 1 = not sure at all, 2 = somewhat sure, 3 = sure, and 4 = very sure. A higher composite score of the five items indicates a greater level of confidence in managing falls than lower scores [33]. Because a fear of falling, balance or walking problems, and previous falls are associated with reduced quality of life in previous studies [34,35,36], participants were also surveyed for changes in health-related quality of life (HRQOL) pre- to post-intervention using the Center for Disease Control’s Healthy Days Core Module [37]. The Healthy Days modules assesses HRQOL using self-rated general health on a five-point scale, with 1 = excellent, 2 = very good, 3 = good, 4 = fair, and 5 = poor and an estimate of the total number of unhealthy days based on the number of days during the past 30 days in which they experienced poor physical health or mental health [37].

### 2.5. Data Analysis

Descriptive statistics (n, % or n, mean, standard deviation) were calculated for participant demographic variables. Results of the Shapiro–Wilk test for normality for all continuous variables (i.e., self-rated health and FES scores) indicated that paired *t*-test analyses were appropriate to compare baseline and final self-assessed general health, healthy days, and FES scores for completers within targeted and non-targeted areas. A significant result for this test suggests that the two matched variables are reliably different from each other (e.g., pretest scores are significantly different from posttest scores). Significance was set at *p* < 0.05, 2-tailed. Cohen’s d was calculated as effect size with the following interpretations: small (d = 0.2), medium (d = 0.5), and large (d = 0.8) differences between groups. All analyses were performed using SPSS (version 27, IBM Corporation, Armonk, NY, USA). The researchers obtained the required approval from the North Texas Regional Institutional Review Board.

## 3. Results

### 3.1. Program Implementation

Implementation data are presented in Table 2 by targeted and non-targeted areas. AMOB classes were implemented at 24 unique implementation sites and 19 postal codes, including 8 (42.1%) of the 13 targeted areas identified by partners. Over half (52%) of the classes were held at sites in targeted areas and had an average enrollment of 12 participants, compared to non-targeted area classes that had an average enrollment of 14 participants. Over one-third (37%) of participants were enrolled in targeted area classes. Seven classes, with six in targeted areas, were discontinued at the onset of the COVID-19 pandemic in March 2020 and unable to complete all eight sessions. After excluding these classes from the sample, the average participant completion rate was 65% for targeted area classes and 70.5% for non-targeted area classes.

### 3.2. Characteristics of AMOB Completers

Demographic characteristics are presented in Table 3 for targeted and non-targeted areas. N = 188 older adults completed five of eight sessions of the AMOB class, ranging in ages from 54 to 94 years with an average age of 76.37 ± 10.36. Most participants identified as female (n = 150, 80%), White-Non-Hispanic (n = 163, 86.7%), and speaking English as their preferred language (n = 143, 97%). Over one-third (n = 66, 35.1%) of the participants completed classes in targeted areas.

### 3.3. Outcome Variables

Changes in FES and HRQOL from baseline to post-intervention were evaluated to determine the efficacy of AMOB in community-dwelling older adults. Table 4 provides detailed information for paired *t*-tests for self-assessed general health and FES for targeted and non-targeted areas. As shown in Table 4, there were significant improvements in overall falls efficacy for completers in targeted areas (t = −4.58; *p* < 0.001) and non-targeted areas (t = −7.30; *p* < 0.001), both with large effect sizes as seen by Cohen’s d. Individual item results showed significant moderate improvement. No statistical differences (*p* > 0.05) were observed for the self-assessment of HRQOL measures for completers in targeted versus non-targeted areas.

## 4. Discussion

The purpose of this study was to examine outcomes of a partnership among an academic institution, government agency, community non-profit organizations, and emergency management services to implement a falls prevention training program using an AFHS approach to increase falls prevention in North Texas in target and non-targeted areas. In this study, implementation through strategic partnerships resulted in twenty-seven AMOB classes delivered over a twelve-month period, with almost one-third of the classes delivered in targeted areas.

In this study, we used the AFHS process of identifying gaps in services to target expansion of an evidence-based program for older adults, which resulted in half of classes being delivered in targeted areas. However, classes in non-targeted areas yielded higher completion rates. Not only were there lower completion rates in targeted area classes, but also most of the discontinued classes at the onset of COVID-19 were in target areas. Such differences could be the result of retention challenges described by other studies that identified social, economic, and health and cognitive barriers to completion for community-dwelling high-risk older adults [38,39,40]. Based on these studies, an implementation strategy should consider increasing awareness through widespread promotional efforts; partnerships with trusted community members, hospitals, and organizations within high risk communities; and participant support addressing individual barriers, such as phone call reminders and transportation vouchers [40,41,42,43]. Recruitment can also be supported through resource-intensive but high-yielding efforts to build local capacity for a variety of potentially beneficial programs, including online and in-person options [44,45]. Completion rates were also affected by the COVID-19 pandemic that forced immediate closure of active AMOB classes that were primarily in “hot spots”.

Based on analysis of pre- and post-measures, older adults completing the program in both targeted and non-targeted areas reported significant improvements in falls efficacy from pre- to post-intervention. These results are consistent with other studies demonstrating AMOB completion is associated with increased knowledge and skills in falls risk reduction activities and improved self-efficacy for high-risk older adults, such as those in underserved areas, living with a disability, or at an advanced age (age 85 years or older) [46,47,48,49]. Furthermore, a systematic review found evidence supporting AMOB’s effect on falls efficacy in addition to reducing the fear of falling [50]. For this study, post-intervention measures were collected at the completion of the eight-week class. Other researchers have shown modest but lasting effects of AMOB on improved falls efficacy [31,33,50]. Healy et al. [31] reported a sustained increase in falls efficacy at six and twelve months post-intervention. Therefore, participating in supplemental interventions after completing AMOB can help sustain fall efficacy over time.

However, there were no significant effects of the intervention on quality of life (HRQOL items). The association between AMOB and HRQOL has not been supported in previous studies that found AMOB was less effective in improving HRQOL compared to its positive effect on falls efficacy, fear of falling, and activity levels [33,51]. These results may also be due to recruitment of a healthier, low-risk population, as seen in the sample’s average falls efficacy at baseline being at mid-range, whereas a high-risk population may indicate lower levels of falls efficacy. Noh et al.’s [52] study of over 32,000 older adults age 65 found a significant relationship between high risk of falls injury and poor HRQOL. Chen et al. [53] found similar AMOB implementation results in which most enrollees were less likely to have sustained a fall. The effect of falls interventions on HRQOL is mixed, with other studies demonstrating significant improvements in physical rather than mental or psychological dimensions of HRQOL [54,55,56]. It is also possible that falls interventions implemented over longer periods could be beneficial to quality of life, as seen King et al.’s [57] study in which a 12-month community-based physical activity intervention improved physical domains of HRQOL. Therefore, implementing a falls risk intervention should consider its effect on specific factors that can contribute to quality of life and the opportunity to extend beneficial effects over time through complementary programs.

There were limitations associated with this study. The onset of the COVID-19 pandemic in March 2020 resulted in missing data and the immediate discontinuation of multiple active classes. A second limitation was the method of collecting self-reported data that required participants to recall occurrences, which may have introduced recall bias. Another limitation was the in-person group delivery model for AMOB that is dependent on multiple factors (e.g., available resources, illness); therefore, intervention delivery may need to be tailored for different goals, preferences, available funding, target populations, and partnerships. Despite these limitations, this study is among a few studies to examine the effect of AMOB implementation among specific targeted zip codes using the AFHS framework.

## 5. Conclusions

Novel methods of delivering community-based health promotion interventions can help administer limited resources towards improving enrollment, retention, and participant outcomes. Using the AFHS approach of implementing falls risk prevention training in targeted areas can be an effective strategy to increase participation and improve falls efficacy for program completers, although the location of training is one factor of many that can influence participation. Stakeholder groups that represent at-risk communities should be involved in coordinated efforts to recruit participants and address financial and transportation barriers to participation. Program adaptations for home-based or virtual delivery are needed to continue falls prevention training for older adults with barriers to access and completion, especially during the continued pandemic. Since the pandemic, AMOB and other falls prevention activities have been adapted for synchronous online participation, providing additional options for types and modalities. These can be considered in developing an implementation strategy that incorporates complementary falls risk prevention interventions and supports continued beneficial outcomes for participants.

## Figures and Tables

**Table 1:** Age-Friendly Health System 4Ms components of A Matter of Balance (AMOB) model.

What Matters	Medications	Mobility	Mentation
AMOB targets community-dwelling older adults who are concerned about falls, are becoming socially isolated to avoid falling and are interested in improving their flexibility, balance, and strength.	One session of AMOB includes the role medications play in fall risk. Participants learn the importance of asking their physicians about medications and their own role in taking them appropriately.	The eight-session curriculum includes exercises to improve strength and balance.	During the sessions, a supportive network of peers is developed. The structured activities include group discussion, problem-solving, skill building, assertiveness training, videos, and sharing practical solutions.

Note. Adapted from Evidence Based Leadership Council & National Association of Area Agencies on Aging. (n.d.). Crosswalk: Evidence-based Leadership Council Programs & the 4Ms. Washington, D.C: Aging and Disability Business Institute.

**Table 2:** Descriptive data of AMOB implementation, July 2019 to June 2020.

Implementation Descriptors	Targeted Areas	Non-Targeted Areas
	N (%)	N (%)
Classes (N = 27)	14 (51.9)	13 (48.1)
Postal codes (N = 19)	8 (42.1)	11 (57.9)
Implementation sites (N = 24)	12 (50.0)	12 (50.0)
Participants enrolled (N = 354)	172 (48.6)	182 (51.4)
Average enrollment per class	12.3	14.0
Participants enrolled, adjusted (N = 275)	102 (37)	173 (63)
Participants completed (N = 188)	66 (35.1)	122 (64.9)
Participant completion rate, adjusted	64.7%	70.5%

Note: The completion rate was calculated with an adjusted enrollment total due to early closure of seven classes that were unable to implement all 8 sessions at the onset of the COVID-19 pandemic.

**Table 3:** Demographic characteristics of participants completing (AMOB).

Characteristic		All (n = 188)	Targeted Areas (n = 66)	Non-Targeted Areas (n = 122)
		N (%)	N (%)	N (%)
Sex	Female	150 (79.8)	53 (80.3)	97 (79.5)
	Male	38 (20.2)	13 (19.7)	25 (20.5)
Age	50–59	1 (0.5)	0 (0.0)	1 (0.8)
	60–69	27 (14.4)	7 (10.6)	20 (16.4)
	70–79	81 (43.1)	24 (36.4)	57 (46.7)
	80–89	60 (31.9)	23 (34.8)	37 (30.3)
	90+	18 (9.6)	11 (16.7)	7 (5.7)
Race	White/Caucasian	163 (86.7)	56 (29.8)	107 (87.7)
	Black/African American	16 (8.5)	8 (4.3)	8 (6.6)
	Asian	1 (0.5)	0 (0.0)	1 (0.8)
	American Indian/Alaska Native	2 (1.1)	0 (0.0)	2 (1.6)
Ethnicity	Not Hispanic or Latino	174 (92.6)	63 (33.5)	111 (91.0)
	Hispanic or Latino	12 (6.4)	3 (1.6)	9 (7.4)
Primary Language	English	183 (97.3)	64 (34.0)	119 (97.5)
	Spanish	2 (1.1)	1 (0.6)	1 (0.8)

**Table 4:** Results of the paired sample *t*-test for healthy days and falls efficacy scale.

Item	Participant Group	N	Baseline	Post-Intervention	t	d
Falls Efficacy Scale (FES)						
Total Score	Targeted areas	45	14.4 (±3.85)	16.00 (±3.08)	−4.58 ***	0.68
	Non-Targeted areas	87	13.9 (±3.78)	16.18 (±3.21)	−7.30 ***	0.78
I can find a way to get up if I fall	Targeted areas	51	2.82 (±0.099)	3.08 (±0.89)	−2.64 *	0.37
	Non-Targeted areas	89	2.85 (±1.01)	3.27 (±0.85)	−4.35 *	0.46
I can find a way to reduce falls	Targeted areas	44	3.11 (±0.81)	3.39 (±0.62)	−2.21 *	0.33
	Non-Targeted areas	86	2.78 (±0.87)	3.35 (±0.68)	−6.82 **	0.73
I can protect myself if I fall	Targeted areas	49	2.39 (±0.93)	2.80 (±0.91)	−3.22 **	0.46
	Non-Targeted areas	88	2.50 (±0.92)	2.94 (±0.89)	−4.71 **	0.50
I can increase my physical strength	Targeted areas	47	3.09 (±0.86)	3.38 (±0.74)	−2.84 **	0.41
	Non-Targeted areas	90	3.00 (±0.90)	3.40 (±0.75)	−4.64 **	0.49
I can become more steady on my feet	Targeted areas	50	2.84 (±0.95)	3.10 (±0.84)	−2.95 **	0.42
	Non-Targeted areas	90	2.88 (±0.85)	3.29 (±0.74)	−4.68 **	0.49
**HRQOL**						
General Health	Targeted areas	51	2.78 (±1.57)	2.49 (±0.92)	1.50	
	Non-Targeted areas	94	3.07 (±1.31)	2.85 (±1.04)	1.60	
Unhealthy days	Targeted areas	45	2.13 (±5.32)	2.84 (±7.27)	−0.63	
	Non-Targeted areas	73	5.23 (±9.57)	3.64 (±7.33)	1.45	

Note: * *p* < 0.05, ** *p* < 0.01, *** *p* < 0.001; Interpretation for d: small (d = 0.2), medium (d = 0.5), and large (d = 0.8) effect size.

## Data Availability

Not applicable.
